# Remote Sensing Image Change Detection Based on NSCT-HMT Model and Its Application

**DOI:** 10.3390/s17061295

**Published:** 2017-06-06

**Authors:** Pengyun Chen, Yichen Zhang, Zhenhong Jia, Jie Yang, Nikola Kasabov

**Affiliations:** 1 College of Information Science and Engineering, Xinjiang University, Urumuqi 830046, China; m13039440466_1@163.com (P.C.); WQLemp0422@163.com (Y.Z.); 2 Institute of Image Processing and Pattern Recognition, Shanghai Jiao Tong University, Shanghai 200400, China; Jieyang@sjtu.edu.cn; 3 Knowledge Engineering and Discovery Research Institute, Auckland University of Technology, Auckland 1020, New Zealand; nkasabov@aut.ac.nz

**Keywords:** change detection, nonsubsampled contourlet transform, Hidden Markov Tree model, NSCT-HMT model, FLICM

## Abstract

Traditional image change detection based on a non-subsampled contourlet transform always ignores the neighborhood information’s relationship to the non-subsampled contourlet coefficients, and the detection results are susceptible to noise interference. To address these disadvantages, we propose a denoising method based on the non-subsampled contourlet transform domain that uses the Hidden Markov Tree model (NSCT-HMT) for change detection of remote sensing images. First, the ENVI software is used to calibrate the original remote sensing images. After that, the mean-ratio operation is adopted to obtain the difference image that will be denoised by the NSCT-HMT model. Then, using the Fuzzy Local Information C-means (FLICM) algorithm, the difference image is divided into the change area and unchanged area. The proposed algorithm is applied to a real remote sensing data set. The application results show that the proposed algorithm can effectively suppress clutter noise, and retain more detailed information from the original images. The proposed algorithm has higher detection accuracy than the Markov Random Field-Fuzzy C-means (MRF-FCM), the non-subsampled contourlet transform-Fuzzy C-means clustering (NSCT-FCM), the pointwise approach and graph theory (PA-GT), and the Principal Component Analysis-Nonlocal Means (PCA-NLM) denosing algorithm. Finally, the five algorithms are used to detect the southern boundary of the Gurbantunggut Desert in Xinjiang Uygur Autonomous Region of China, and the results show that the proposed algorithm has the best effect on real remote sensing image change detection.

## 1. Introduction

Image change detection is the process of identifying changes in land cover through analyzing remote sensing images acquired in the same geographical location at different times [1]. It is widely used in the fields of video surveillance [2], medical diagnosis [3], land use [4], and natural disaster detection [5]. Changes in land use and oasis coverage have a direct impact on the human environment and ecological processes. Information regarding changes in land use and coverage are important for natural resource management and scientific decision-making [6,7], and are a critical component of research in the areas of the environment, forestry, hydrology, agriculture, geography, and ecology. Using remote sensing images for land use and oasis cover change detection can quickly and accurately obtain change information and development trends. However, the rapid updating of land cover data has become a technical problem in the fields of remote sensing and geographic information systems. Methods of quickly and accurately determining changes based on geographical data, and unsupervised techniques for extracting the changes in objects from an image are greatly significant for rapidly updating land use and oasis cover images [8].

Li et al. proposed a multi-scale image change detection algorithm based on Markov Random Field (MRF) information fusion [9]. They introduced the MRF model into remote sensing image change detection. The MRF model describes the state dependent relationship between adjacent image coordinates and expresses the local statistical characteristics of the image. Do et al. proposed the contourlet hidden Markov tree (HMT) model, which was based on the implicit Markov model in the wavelet domain [10]. The dependency between the coefficients was expanded from intra-scale to inter-scale. Gong et al. proposed a SAR image change detection algorithm based on improved MRF energy function and fuzzy clustering (MRF-FCM) [11]. The algorithm described in [9,10] greatly improves detection accuracy; however, it uses the iterative optimization strategy. If the algorithm is directly used for change detection, it involves a large calculation with low efficiency. Although the algorithm proposed in [11] has reduced complexity, the change detection results are still vulnerable to the influence of the clutter noise because the relevant context is absent. Hesen et al. proposed an unsupervised change detection algorithm based on the nonsubsampled contourlet and fuzzy C-mean clustering (NSCT-FCM) algorithm [12]. The efficiency of the algorithm is greatly improved, but the detection accuracy is low. Pham et al. proposed a pointwise approach to detect land-cover changes between two SAR images to capture the image’s significant contextual information (PA-GT) [13]. Yousif et al. proposed a SAR image change detection algorithm based on the principal componenet analysis (PCA) algorithm and the nonlocal means (NLM) denoising algorithm [14].

With the development of remote sensing technology, change detection in remote sensing image has become more and more important. The algorithms that are used to detect SAR images and multi-spectral images change should be different, because the noise is significantly different from SAR and optical image data, but many algorithms can be used to detect both the synthetic aperture radar images and the optical images. Yetgin et al. proposed a novel technique for unsupervised change detection of multi-spectral satellite images using Gaussian mixture model (GMM), local gradual descent, and k-means clustering [15]. Celik et al. proposed an unsupervised change detection method for satellite images which conducted on the synthetic aperture radar images and the optical images [16]. Ma et al. proposed an unsupervised change detection method based on an improved rough fuzzy c-means clustering method (SRFPCM) for synthetic aperture radar and optical remote sensing images [17]. There are also many similar algorithms in the literature [18,19,20,21,22].

In order to reduce the influence of noise on the detection results, we propose a remote sensing image change detection algorithm based on the non-subsampled contourlet transform and the Hidden Markov Tree (NSCT-HMT) model. First, the ENVI software is used to calibrate the original remote sensing images. After that, the difference map is obtained using mean value method, and then the NSCT-HMT model is used to denoise the difference image. The Fuzzy Local Information C-means (FLICM) algorithm is then used to classify the denoised images. The experimental results show that the proposed method is more accurate than MRF-FCM, NSCT-FCM, PA-GT and PCA-NLM algorithms. Finally, the algorithms are applied to detect oasis coverage on the southern boundary of the Gurbantunggut Desert in the Xinjiang Uygur Autonomous Region of China.

## 2. Image Change Detection with NSCT-HMT Model

The ENVI software is used to calibrate the original remote sensing images. After calibration, the images are subjected to decibel transformation, prior to the operation to generate the difference map. In this paper, all parameter values are set the same for all our data.

### 2.1. Generation of the Difference Image

The ways to generate a difference map include the difference method and ratio method, and both can effectively construct difference images from optical remote sensing images, but the difference method is sensitive to image quality and the spectral characteristics of the objective conditions, resulting in a high rate of missed detection and false positives. The ratio method includes the logarithmic ratio method and mean ratio method. Among them, the advantage of the logarithmic ratio method is the ability to convert multiplicative speckle noise to additive noise, and the background information of the difference image obtained by the logarithmic transformation is relatively flat. The disadvantages are that the logarithmic ratio algorithm compresses the variation range of the difference image, and has the characteristics of enhancing the low intensity pixels to weaken the high intensity pixels, and cannot reflect the real change trends to the maximum extent [23]. The mean ratio method, on the other hand, does not have such problems. The mean value rule can effectively enhance the contour of the change area and changes in a small area, and can also prevent the loss of change information. In order to take full account of the relevant context, the difference map constructed by the mean value ratio depends only on relative changes in image intensity. It can truly reflect the changes in the region and retain more details. Therefore, in this paper, we use the mean ratio method proposed by Inglada et al. to generate the difference image [24].

Assume F and G are images of the same location taken at different times and the size of these images are *r* pixels × *c* pixels, and they have been calibrated by ENVI.

The mean-ratio operation formula is as follows:

(1) $$X_{m}(i,j)=1-\min\left(\frac{\mu_{1}(i,j)}{\mu_{2}(i,j)},\frac{\mu_{2}(i,j)}{\mu_{1}(i,j)}\right)$$

where *μ*_1_ (*i, j*) and *μ*_2_ (*i, j*) respectively represent the local mean value of F and G at the point (*i*, *j*) in the window size of *ω* × *ω*, *ω* = 1, 3, 5, 7, ...

### 2.2. A Denoising Method Based on the NSCT-HMT Model

After obtaining the mean difference map, this paper uses the non-sampling contourlet HMT (NSCT-HMT) model proposed by Wang et al. to denoise the difference image [25]. The original NSCT-HMT model is mainly used to process the optical images. In this paper, we apply it to remote sensing image denoising. The overview of the basic rationale of the adopted methodologies:

1. The mean difference map was dealt with the NSCT decomposition;
2. After the NSCT decomposition, the coefficients of NSCT are fitted by Gauss mixture model;
3. Using the HMT model to estimate the parameters;
4. The Monte-Carlo method is used to generate random white noise images and balance the variance of the image in the NSCT domain;
5. The EM algorithm is used to estimate the NSCT coefficient of the denoised image;
6. Using the NSCT synthesis to get denoised image.

#### 2.2.1. NSCT Transform and the Hidden Markov Model

Da et al. proposed a kind of non-sampling contourlet transform (NSCT) that was based on the contourlet transform [26]. The NSCT does not have a sampling process in the image decomposition and reconstruction process, and it is composed of two-dimensional non-sampled pyramid filter banks and non-down-sampling filter banks. The result of this treatment is that the NSCT transform has translation invariance in addition to being multi-resolution, multi-scale, and anisotropic. Although the NSCT coefficients do not obey a Gauss distribution, they follow a zero mean mixed Gauss distribution. The NSCT coefficients can be modeled using a mixture of two Gauss functions. They have the characteristics of persistence and aggregation, and have a strong dependence on the neighborhood coefficient. These NSCT characteristics have laid the foundation for the establishment of the Markov model in the NSCT domain (NSCT-HMT). After obtaining the mean difference map, three layers of NSCT decomposition was performed. The number of sub-bands in each layer is 4, 4 and 8.

The hidden Markov model (HMT) is a double stochastic process consisting of a hidden Markov chain with a certain number of states and a set of random functions. It can be divided into a hidden layer and an observation layer. The hidden layer cannot be directly observed, and includes a certain number of states. The observation layer (actual observation) can have some observations corresponding to the state. The observation vector is generated by a sequence of states with probability density distribution. It is used to express various states by a probability density distribution.

#### 2.2.2. Establishment of NSCT-HMT Model

In theory, a signal needs to be accurately represented by a series of Gaussian distributions. However, in the actual image processing application, it is possible to use the 2~4 Gaussian mixture to approximate the image [27,28]. The coefficients of NSCT can be modeled using a mixture of two Gauss functions. Formulas (2) and (3) are used to build the coefficients of the Gauss mixture model [25]:

(2) $$f_{N_{i}}(N_{i})=\sum_{m=1}^{n}f_{s_{i}}(m)f_{N_{i}|s_{i}}\;(N_{i}/s_{i}=m)$$

(3) $$f_{N|s_{i}}(N_{i}|s_{i}=m)=\frac{1}{\sqrt{2\pi \sigma_{im}^{2}}}\exp[-\frac{{\left(N_{i}-\mu_{im}\right)}^{2}}{2\sigma_{im}^{2}}]=g(N_{i};\mu_{im};\sigma_{im}^{2})$$

where *N_i_* represents the coefficient of a certain subband in each layer. *s_i_* indicates in which state the coefficients are selected. *fs_i_*(*m*) is the probability distribution function of the large and small states, and $\sum_{m=1}^{n}f_{s_{i}}(m)=1$; $f_{N_{i}|s_{i}}(N_{i}|s_{i}=m)$ represents the probability distribution density function corresponding to the Gauss model when the coefficients are in a specific state. $\mu_{im}$ and $\sigma_{im}^{2}$ represent the mean and variance of the Gauss distribution, respectively.

The probability distribution function of $N_{1}$ in each direction sub-band node can be expressed as $P_{N_{1}}(m)$, where m is the number of hidden states. State transition probabilities can be expressed as $\varepsilon_{i,\rho (i)}^{m,n}=f(s_{i}=m|s_{\rho }(i)=n)$.

The above parameters are represented by a formal $\theta_{N-HMT}$ of four tuples [25]:

(4) $$\theta_{N-HMT}=\{(P_{N_{1}}(m),\varepsilon_{i,\rho (i)}^{m,n},\sigma_{i,m}^{2},\mu_{i,m})|i=1,2,\ldots ,p;m,n=1,2,\ldots ,M\}$$

where *P* represents the number of coefficients and *M* represents the number of states. The NSCT-HMT model can be represented by the four tuple of parameters as shown in Formula (4).

#### 2.2.3. Image Denoising Based on NSCT-HMT Model

In order to calculate of the model parameters, we use the HMT-EM algorithm proposed by Fan et al. to estimate the parameters [29], and the local optimal solution is obtained by iteration. A noisy image can be understood as the superposition of original image information and noise. If we assume that the NSCT coefficient of the difference image is *y*, then *y* is composed of the NSCT coefficient *x* of the original image and the NSCT coefficient *n* of the noise. So the image denoising problem can be transformed into the estimation of the NSCT coefficient *x* of the original image in the case of the NSCT coefficient *y* of the noisy image.

Image denoising based on NSCT-HMT model is as follows:

1. By Equation (4), the parameters of the model can be expressed as:
  (5) $$\theta_{N-\mathrm{HMT}}=\left\{P_{N_{1}}(m),\varepsilon_{i,\rho (i)}^{m,n},\sigma_{i,m}^{2},\mu_{i,m}\right\}$$
2. The Monte-Carlo method proposed by Crouse et al. is used to generate random white noise images and balance the variance of the image in the NSCT domain [30], and then the noise variance of the coefficient is estimated in the noise image model. The variance of the noise coefficient can be expressed as ${\left(\sigma_{\left(j,k,i\right)}^{\left(\mathrm{noise}\right)}\right)}^{2}$. The coefficient model of the original image can be obtained by subtracting the noisy image’s variance of the noise coefficient of the NSCT-HMT model [26]:
  (6) $${\left(\sigma_{(j,k,i),m}^{x}\right)}^{2}=[{\left(\sigma_{(j,k,i),m}^{y}\right)}^{2}-{\left(\sigma_{\left(j,k,i\right)}^{\left(\mathrm{noise}\right)}\right)}^{2}]$$
  where *j*, *k*, *i* respectively refer to the scale, direction, and coefficient. *m* indicates the hidden state of the NSCT coefficient.
3. The NSCT-HMT parameter model of the denoised image can be expressed as:
  (7) $$\theta_{N-\mathrm{HMT}-x}=\left\{P_{N_{1}}(m),\varepsilon_{i,\rho (i)}^{m,n},{\left(\sigma_{i,m}^{\left(x\right)}\right)}^{2},\mu_{i,m}\right\}$$
  The above parameters are used to estimate the NSCT coefficients of the denoised image. When the state S*_j,k,i_* is constant, the NSCT coefficients obey the Gauss distribution. It is assumed that the noise is Gauss white noise with a mean value of 0, and it is independent of the NSCT coefficient. Then the NSCT coefficient of the Bayes denoised image [26] can be represented as:
  (8) $$E[x_{j,k,i}|y_{j,k,i},\theta_{N-\mathrm{HMT}-x},S_{j,k,i}]=\frac{{\left(\sigma_{(j,k,i),m}^{\left(x\right)}\right)}^{2}}{{\left(\sigma_{(j,k,i),m}^{\left(x\right)}\right)}^{2}+{\left(\sigma_{(j,k,i),m}^{\left(n\right)}\right)}^{2}}\times y_{j,k,i}$$
4. The conditional probability obtained by the EM algorithm [26] can be expressed as:
  (9) $$p(S_{j,k,i}=m|y_{j,k,i},\theta_{N-\mathrm{HMT}-x})$$
5. The NSCT coefficient of the denoised image can be estimated as:
  (10) $$E[x_{j,k,i}|y_{j,k,i},\theta_{N-\mathrm{HMT}-x}]=\sum_{m}p(S_{j,k,i}=m|y_{j,k,i},\theta_{N-\mathrm{HMT}-x})\times \frac{{\left(\sigma_{(j,k,i),m}^{\left(x\right)}\right)}^{2}}{{\left(\sigma_{(j,k,i),m}^{\left(x\right)}\right)}^{2}+{\left(\sigma_{(j,k,i),m}^{\left(n\right)}\right)}^{2}}\times y_{j,k,i}$$

### 2.3. FLICM Clustering

In this paper, we use the FLICMC clustering algorithm proposed by Krindis et al. [31]. This algorithm modifies the objective function of the traditional FCM algorithm, and introduces the fuzzy factor *G_ki,_*:

(11) $$G_{ki}=\sum_{j=N_{i}}\frac{1}{d_{ij}+1}{\left(1-\mu_{kj}\right)}^{n}{\left\|x_{j}-\nu_{k}\right\|}^{2}$$

where $d_{ij}$ is a spatial euclidean distance between the *i* pixel and the neighborhood *j* pixel. $\mu_{kj}$ indicates the degree of ownership of the *j* pixel to class *k*. $x_{j}$ represents the neighborhood pixels near the center *i* pixel in the local window. $v_{k}$ is the clustering center of class *k*.

The objective function of the FLICM algorithm is shown below:

(12) $$J_{m}=\sum_{i=1}^{N}\sum_{k=1}^{c}\left[\mu_{ki}^{n}\|x_{i}-{v_{k}\|}^{2}+G_{ki}\right]$$

where $x_{i}$ is the center pixel of local window. $v_{k}$ and $\mu_{kj}$ respectively represent the class k clustering centers and fuzzy membership matrix. They can be expressed as:

(13) $$v_{k}=\frac{\sum_{i=1}^{N}\mu_{ki}^{n}x_{i}}{\sum_{i=1}^{N}\mu_{ki}^{n}}$$

(14) μki=1∑j=1c(‖xi−vk‖2+Gki‖xi−vk‖2+Gji)1(n−1)

### 2.4. Implementation Steps

The implementation steps are as follows:

Step 1: The original remote sensing images are calibrated by ENVI software;
Step 2: The difference image is obtained by using Equation (1) on the calibrated images;
Step 3: The NSCT transform is used to transform the difference image, and the transformed coefficients are modeled using a hidden Markov tree. Equation (6) is used to obtain the coefficients of the original image. Finally, Equation (10) is used to estimate the NSCT coefficients of the denoised image.
Step 4: The Inverse NSCT transform is used to obtain the difference image after denoising.
Step 5: After the above treatment, Equation (12) is used to cluster the difference map, and the final change detection results are obtained.

**Figure 1:**
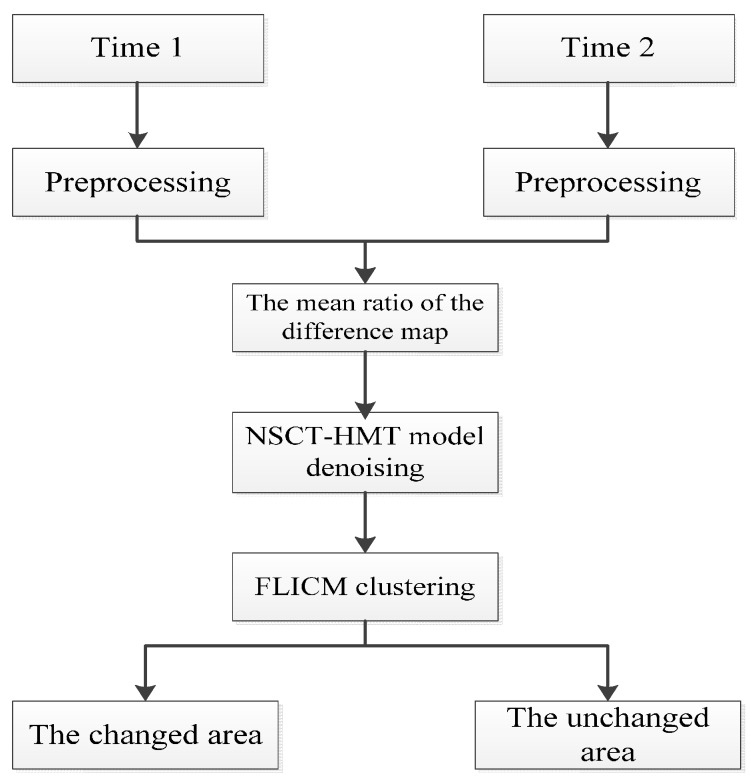
The algorithm flowchart.

The algorithm flow is shown below as Figure 1.

## 3. SAR Image Change Detection

### 3.1. The Selection of the Window Size for SAR Image

To illustrate the effect of window size on change detection results, we examine the relationship between window size *ω* × *ω* and percentage correct classification (PCC).

**Figure 2:**
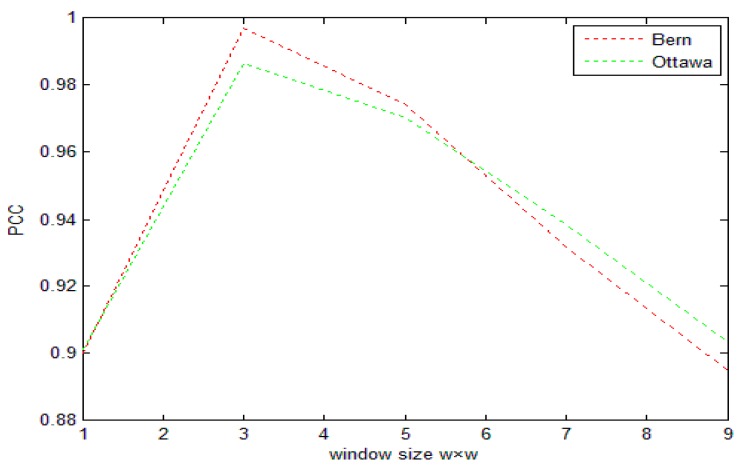
The relationship between window size *ω* × *ω* and PCC.

As can be seen in Figure 2, when the window size is 3 × 3, PCC can get the optimal solution. The reason is that if the selection is too large, the noise removal is relatively clean, but it will lead to the loss of edge and detail information. If selected too small, more details will be retained, but the denoising effect is poor, so for the SAR images in this paper, *ω* = 3.

### 3.2. SAR Image Data Description

In order to verify the effectiveness and to illustrate the practicality of the proposed method, we use two sets of real SAR images in [32]. The reason why we choose these two sets of SAR images is that these two groups of images are well known and often used for comparison.

#### 3.2.1. Real Remote Sensing Image of the Bern Data Set

**Figure 3:**
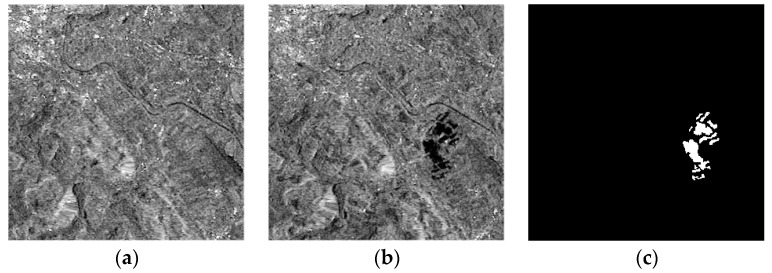
Bern data set: (**a**) Image acquired in April 1999; (**b**) Image acquired in May 1999; (**c**) The reference change image.

The first sets of real SAR image data of Bern, Switzerland were obtained by ERS-2 in April 1999 and May 1999, and are shown below in Figure 3a,b. The images are 301 × 301 pixels in size and have a gray level of 256. Changes are mainly caused by a flood, and the reference map is shown in Figure 3c.

#### 3.2.2. Real Remote Sensing Image of Ottawa Data Set

**Figure 4:**
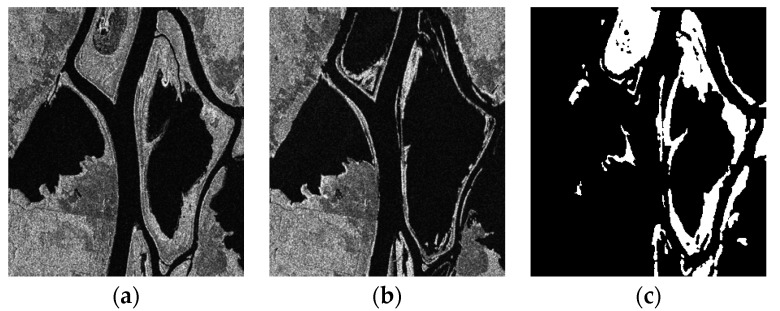
Ottawa data set: (**a**) Image acquired in May 1997; (**b**) Image acquired in August 1997; (**c**) The reference change image.

The second set of real SAR image data from Ottawa, Canada was obtained by Radarsat in May 1997 and August 1997. They are shown below as Figure 4a,b. The images are 290 × 350 pixels and have a gray level of 256. The main change is the surface change caused by flooding, and the reference map is shown in Figure 4c.

### 3.3. SAR Image Data Experimental Results and Analysis

**Figure 5:**
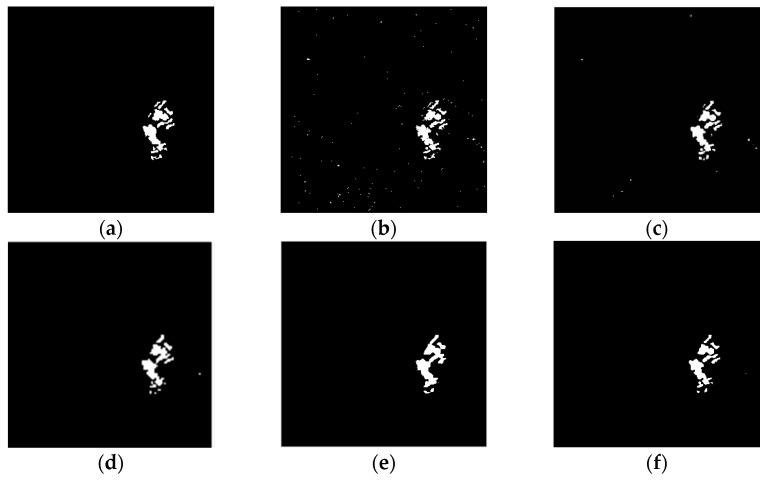
Change detection images obtained for the Bern data set: (**a**) The reference change image; (**b**) NSCT-FCM; (**c**) MRF-FCM; (**d**) PA-GT; (**e**) PCA-NLM; (**f**) Proposed algorithm.

**Figure 6:**
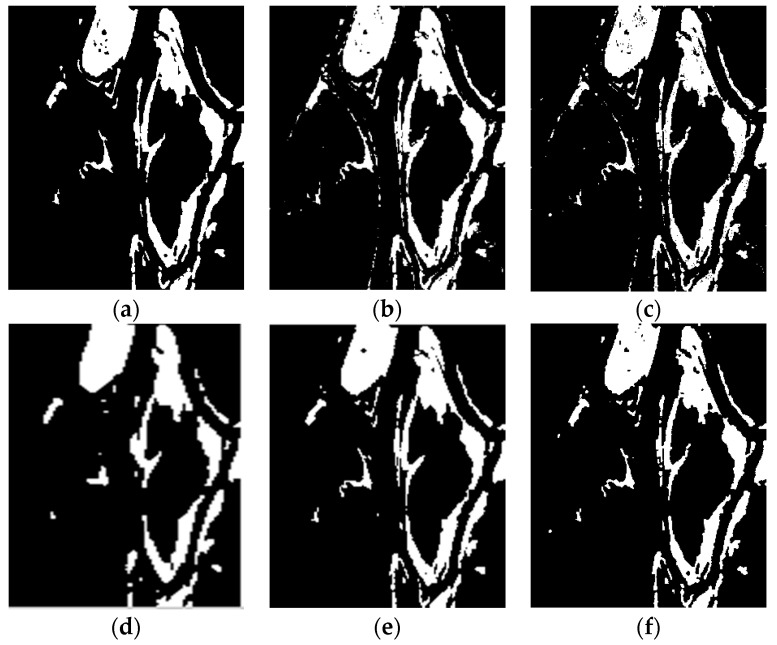
Change detection images obtained for the Ottawa data set: (**a**) The reference change image; (**b**) NSCT-FCM; (**c**) MRF-FCM; (**d**) PA-GT; (**e**) PCA-NLM; (**f**) Proposed algorithm.

To evaluate the effectiveness of the proposed algorithm, we use the above three real remote sensing image data sets for change detection. The algorithm proposed in this paper is compared with the MRF-FCM [11], NSCT-FCM [12], PA-GT [13] and PCA-NLM [15] algorithms. Results of the qualitative analysis are shown in Figure 5 and Figure 6.

As can be seen from Figure 5f, there is no isolated clutter in the resulting graph, which means that the algorithm proposed in this paper can effectively suppress non-changing clutter. It can be seen from Figure 6f that the resulting image effectively suppresses noise effectively and preserves the details of the change.

In order to quantitatively evaluate the performance of the algorithm, we use the MRF-FCM, NSCT-FCM, PA-GT, PCA-NLM and the proposed algorithm to detect change in two groups of data sets, and quantitatively analyze the test results. The evaluation indexes are false negatives (FN), false positives (FP), overall error (OE), percentage correct classification (PCC), the Kappa index and the running time (T) (the running time was obtained by calculating the mean value of 20 runs).

The results are shown in Table 1. As can be seen in Table 1, in comparison with MRF-FCM, NSCT-FCM, PA-GT and PCA-NLM, the proposed algorithm has the lowest number of false positives, the lowest total number of errors, and the highest PCC and the Kappa index. As for the running time, although the proposed algorithm takes a bit longer than the NSCT-FCM, PA-GT and PCA-NLM algorithms, the detection accuracy is much higher than theirs.

### 3.4. The General Applicability of Verification Glgorithm for SAR Image

The five algorithms are used to process the experimental data of 30 groups. The average results are shown in Table 2.

For the running time, this paper takes the experimental data of 30 groups of 300 × 300 pixels images. The data for each group were run 20 times and then averaged. The results of each parameter are shown in Table 2. The following conclusions can be drawn from Table 2:

1. Compared with the previous algorithms, the proposed algorithm can suppress clutter noise better while preserving detailed information from the original image.
2. The proposed algorithm has a faster processing speed than MRF-FCM, but it is a little slower than NSCT-FCM, PA-GT and PCA-NLM. The complexity of the proposed algorithm needs to be improved.
3. The proposed algorithm is suitable to detect SAR images.

## 4. Multi-Spectral Image Change Detection

Due to the fact that SAR image processing is intrinsically complex, with the presence of the speckle noise, it is difficult to analysis SAR images, and the multi-spectral images are easy affected by the presence of cloud cover or different sunlight conditions. The application to SAR and multi-spectral optical data should be different, but there are many algorithms that can be used to detect both the synthetic aperture radar images and the optical images. Just as the algorithms proposed in [13,14,15,16,17,18,19,20]. We are lucky enough to find an algorithm which can be used for both SAR and multi-spectral remote sensing image change detection.

### 4.1. The Selection of the Window Size for Multi-Spectral Remote Sensing Image

**Figure 7:**
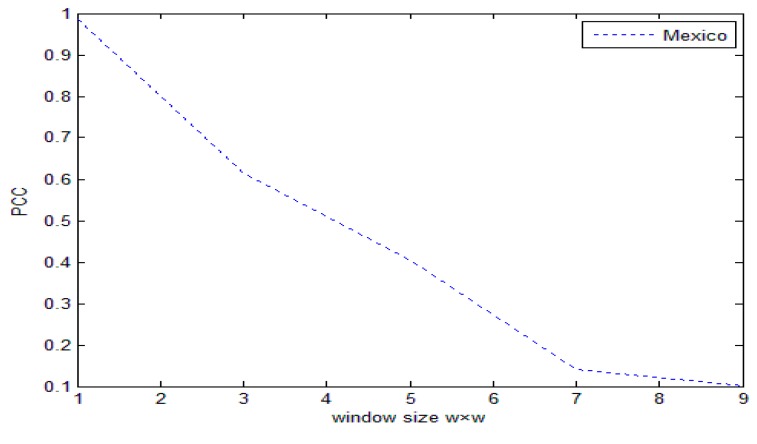
The relationship between window size *ω* × *ω* and PCC.

When the image spatial resolution is 30 m, the relationship between window size *ω* × *ω* and percentage correct classification (PCC) is as follows: as can be seen in Figure 7, when the window size is 1 × 1, PCC can get the optimal solution, so we choose ω = 1 when we are dealing with multi-spectral remote sensing images.

When the window size is 1 × 1, Equation (1) will be changed into:

(15) $$X_{m}(i,j)=1-\min\left(\frac{X_{1}(i,j)}{X_{2}(i,j)},\frac{X_{2}(i,j)}{X_{1}(i,j)}\right)$$

where *X*_1_ (*i, j*) and *X*_2_ (*i, j*) respectively represent the value of the pixel at the point (i,j).

### 4.2. Image Data Description

**Figure 8:**
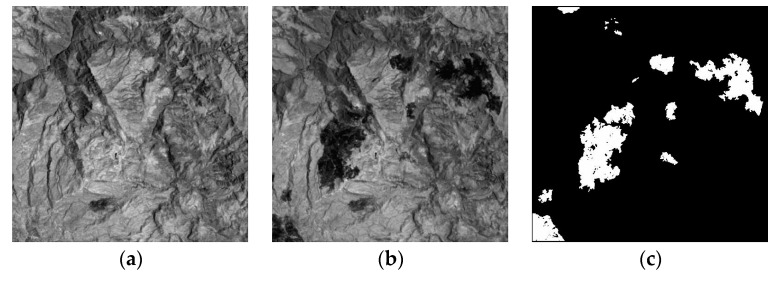
Mexico data set: (**a**) Image acquired in April 2000; (**b**) Image acquired in May 2005; (**c**) The reference change image.

The set of real multi-spectral remote sensing image data are acquired by the Landsat Enhances Thematic Mapper Plus (ETM+) sensor of the Landsat-7 satellite, in an area of Mexico in April 2000 and May 2002. Figure 8a,b shows channel 4 of the 2000 and 2002 images, respectively. The instrument’s pixel resolution is 30. The images are 512 × 512 pixels and have a gray level of 256. The changes were caused by a fire that burned a large part of the vegetation in the test region, and the reference map is shown in Figure 8c.

### 4.3. Multi-Spectral Image Data Experimental Results and Analysis

**Figure 9:**
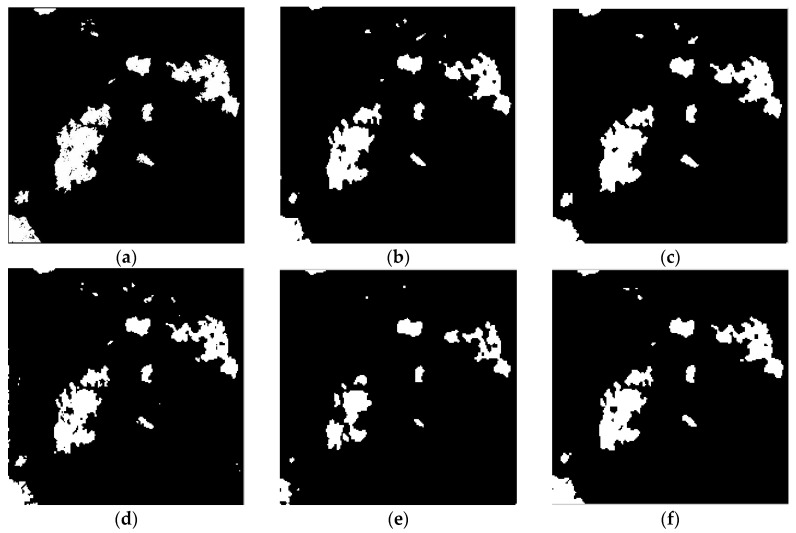
Change detection images obtained for the Mexico data set: (**a**) The reference change image; (**b**) NSCT-FCM; (**c**) MRF-FCM; (**d**) PA-GT; (**e**) PCA-NLM; (**f**) Proposed algorithm.

The evaluation indexes are false negatives (FN), false positives (FP), overall error (OE), percentage correct classification (PCC), the Kappa index and the running time (T). The following conclusions can be drawn from Figure 9 and in Table 3:

1. The PA-GT and PCA-NLM algorithms can achieve good results when dealing with SAR images. But, in dealing with real multi-spectral remote sensing images, the results are poor.
2. The proposed algorithm can be used for multi-spectral remote sensing image change detection.

### 4.4. The General Applicability of Verification Glgorithm for Multi-Spectral Image

The five algorithms are used to process the experimental data of 30 groups. The images are 500 × 500 pixels in size. The average results are shown in Table 4.

The following conclusions can be drawn from Table 4:

1. Compared with the previous four algorithms, the proposed algorithm has a good balance in the number of false negatives and false positives.
2. The proposed algorithm is more suitable to detect multi-spectral images.

## 5. Application

### 5.1. Acquisition of Real Remote Sensing Image

The Gurbantunggut Desert is located in the hinterland of the Junggar basin in northern Xinjiang, China. It is China’s largest fixed and semi-fixed desert, and is located at 44°11′~46°20′ N and 84°31′~90°00′ E. Its acreage is about 4.88 × 10^4^ km^2^. Precipitation occurs mainly in the spring and annually does not reach 150 mm. Its average annual evaporation potential is higher at more than 2000 mm, and the annual average temperature is 6~10 °C.

**Figure 10:**
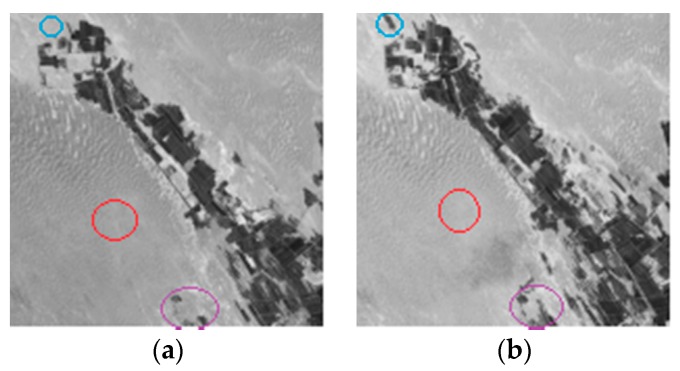
Gurbantunggut data set: (**a**) Image acquired in July 1999; (**b**) Image acquired in July 2007.

We choose a set of real multi-spectral remote sensing image data which are acquired by the Landsat Enhances Thematic Mapper Plus (ETM+) sensor of the Landsat-7 satellite, in an area of Gurbantunggut Desert in July 1999 and July 2007. The instrument’s pixel resolution is 30 m. Figure 10a,b shows channel 3 of the 1999 and 2007 images, respectively. They are registered using ENVI. The images are 570 × 570 pixels, and have a gray scale of 256. The image data is mainly composed of desert, vegetation, and street construction. Changes mainly result from the government and citizens combating desertification and planting vegetation and crops.

### 5.2. Analysis the Change of Oasis Cover

**Figure 11:**
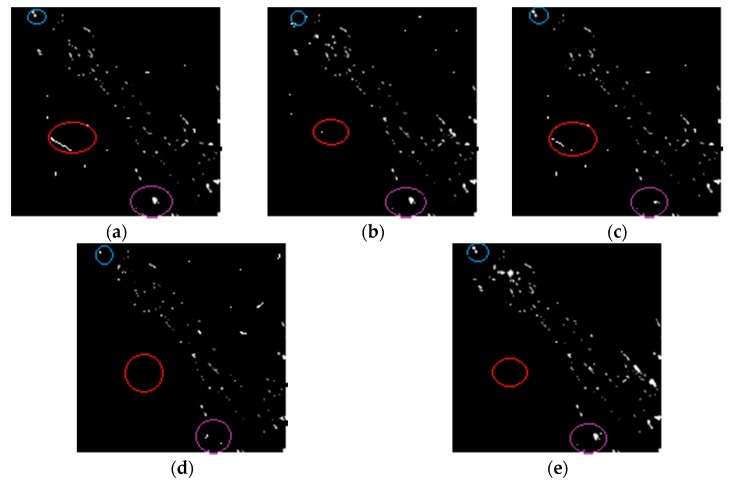
Change detection images obtained for Gurbantunggut data set: (**a**) NSCT-FCM; (**b**) MRF-FCM; (**c**) PA-GT; (**d**) PCA-NLM; (**e**) Proposed algorithm.

The above five algorithms are used to detect the change of Gurbantunggut data set. The results of the change of oasis cover in the study area are shown in Figure 11 and Table 5. The white pixels in Figure 11 represent the oasis cover change.

In the blue and purple areas, parts of the places have indeed changed, but the MRF-FCM and PCA-NLM algorithms did not detect the changed parts. In the red area, there is no change in this area, but NSCT-FCM and PA-GT algorithms have detected changes.

## 6. Conclusions

A remote sensing image change detection algorithm based on the NSCT-HMT denoising model is proposed in this paper. The proposed change detection method is tested for both optical and synthetic aperture radar satellite images. The mean-ratio operation is adopted to obtain the difference image, which will be denosing by the NSCT-HMT model. Then, using the FLICM algorithm, the difference image is divided into the change area and unchanged area. The experimental results show that compared with MRF-FCM, NSCT-FCM, PA-GT and PCA-NLM algorithms, the algorithm proposed in this paper takes full account of the characteristics of the spatial neighborhood. It not only enhances the changes of the regional profile and the small area, but also can effectively suppress the clutter noise, and the final change detection accuracy is improved. Finally, we apply the algorithms to detect the oasis coverage of the southern Gurbantunggut Desert, and the results show that the proposed algorithm has a good detection ability.

In this paper, the multi-spectral optical data sets are acquired by the Landsat-7, so we did not list the relationship between the value of window size and the image spatial resolution in the paper, but we did some experiments, and we found that when the image spatial resolution is lower than 10 m, the value of window size must choose 1 × 1, or using other method such as the different method or log- ratio method, etc., to get the multi-spectral optical different image. There are many places in this paper that need to be improved. For example, how to reduce the complexity of the algorithm and how to further improve the accuracy of the algorithm. In our future investigations, additional work will be conducted on them.

## Figures and Tables

**Table 1:** SAR image change detection results under evaluation by different algorithms.

Data Set	Method Used	*FN*	*FP*	*OE*	*PCC* (%)	*Kappa*	*T* (s)
Bern	NSCT-FCM	168	282	450	99.50	0.8146	8.43
	MRF-FCM	47	364	411	99.55	0.8453	73.91
	PA-GT	195	109	304	99.67	0.8621	10.06
	PCA-NLM	96	267	363	99.61	0.8517	11.29
	Proposed Method	173	103	276	99.69	0.8796	18.43
Ottawa	NSCT-FCM	236	2286	2522	97.51	0.9112	9.36
	MRF-FCM	681	1079	1760	98.26	0.9355	83.81
	PA-GT	1010	747	1757	97.91	0.9203	12.43
	PCA-NLM	781	872	1653	98.37	0.9387	14.08
	Proposed Method	532	833	1365	98.65	0.9498	20.37

**Table 2:** Results of different algorithms for detecting the average change of SAR remote sensing images.

Method Used	$\bar{FN}$/%	$\bar{FP}$/%	$\bar{OE}$/%	$\bar{PCC}$/%	$\bar{Kappa}$	$\bar{T}$/s
NSCT-FCM	10.72	9.86	20.58	79.42	0.8043	8.34
MRF-FCM	5.04	13.42	18.46	81.54	0.8246	73.58
PA-GT	3.23	4.04	7.27	92.73	0.8742	10.02
PCA-NLM	4.21	7.43	11.64	88.36	0.8649	11.04
Proposed Method	2.11	2.73	4.84	95.16	0.8927	17.95

**Table 3:** Multi-spectral image change detection results under evaluation by different algorithms.

Data Set	Method Used	*FN*	*FP*	*OE*	*PCC* (%)	*Kappa*	*T* (s)
Mexico	NSCT-FCM	1030	2402	3432	98.69	0.7418	15.48
	MRF-FCM	1699	724	2423	99.07	0.8143	124.50
	PA-GT	997	1586	2583	99.01	0.7831	20.72
	PCA-NLM	4395	126	4521	98.09	0.5628	23.65
	Proposed Method	1425	671	2091	99.11	0.8417	28.54

**Table 4:** Results of detecting the average change of multi-spectral remote sensing images.

Method Used	$\bar{FN}$/%	$\bar{FP}$/%	$\bar{OE}$/%	$\bar{PCC}$/%	$\bar{Kappa}$	$\bar{T}$/s
NSCT-FCM	5.18	7.93	13.11	86.89	0.7864	15.36
MRF-FCM	4.97	3.82	8.79	91.21	0.8013	122.43
PA-GT	2.83	4.67	7.50	92.50	0.7458	20.45
PCA-NLM	11.59	1.94	13.53	86.47	0.5816	23.51
Proposed Method	2.91	2.23	5.14	94.86	0.8793	27.74

**Table 5:** The running time of five algorithms.

Time	NSCT-FCM	MRF-FCM	PA-GT	PCA-NLM	Proposed Algorithm
T (s)	19.5	154.3	25.7	29.3	35.2
